# Impact of safer supply programs on injection practices: client and provider experiences in Ontario, Canada

**DOI:** 10.1186/s12954-023-00817-7

**Published:** 2023-06-28

**Authors:** Marilou Gagnon, Katherine Rudzinski, Adrian Guta, Rose A. Schmidt, David T. Kryszajtys, Gillian Kolla, Carol Strike

**Affiliations:** 1 School of Nursing, University of Victoria, Victoria, BC Canada; 2 Canadian Institute for Substance Use Research, University of Victoria, 2300 McKenzie Avenue, Victoria, BC V8N 5M8 Canada; 3 School of Social Work, University of Windsor, Windsor, ON Canada; 4 Dalla Lana School of Public Health, University of Toronto, Toronto, ON Canada

**Keywords:** Drugs, Fentanyl, Injection, Overdose, Qualitative research, Safer supply, Substance use

## Abstract

**Objectives:**

Fentanyl has contributed to a sharp rise in the toxicity of the unregulated drug supply and fatal overdoses in Canada. It has also changed injection practices. Injection frequency has increased as a result and so has equipment sharing and health-related risks. The aim of this analysis was to explore the impact of safer supply programs on injection practices from the perspective of clients and providers in Ontario, Canada.

**Methods:**

The data set included qualitative interviews with 52 clients and 21 providers that were conducted between February and October 2021 across four safer supply programs. Interview excerpts discussing injection practices were extracted, screened, coded and then grouped into themes.

**Results:**

We identified three themes, each theme corresponding to a change in injection practices. The first change was a decrease in the amount of fentanyl used and a decrease in injection frequency. The second change involved switching to injecting hydromorphone tablets instead of fentanyl. Finally, the third change was stopping injecting altogether and taking safer supply medications orally.

**Conclusion:**

Safer supply programs can contribute to reducing injection-related health risks in addition to overdose risks. More specifically, they have the potential to address disease prevention and health promotion gaps that stand-alone downstream harm reduction interventions cannot address, by working upstream and providing a safer alternative to fentanyl.

## Introduction

Canada is in the midst of a devastating overdose crisis that has been further exacerbated by the COVID-19 pandemic and, in particular, the subsequent increased toxicity of the drug supply [1]. Between January 2016 and June 2022, close to 33,000 people died from an overdose in Canada, with the provinces of British Columbia, Ontario, and Alberta accounting for 88% of those deaths [2]. Fentanyl, and to a lesser extent fentanyl analogues, have contributed to a sharp rise in the toxicity of the unregulated drug supply and fatal overdoses [2]. Between January and June 2022, fentanyl was detected in 76% of overdose deaths in Canada [2]. In Ontario, fentanyl is involved in 88% of fatal overdoses [3]. In Toronto, this number rises to 93% [3].

Scaling-up interventions such as naloxone distribution programs, supervised consumption services, and opioid agonist treatment have averted thousands of overdose-related deaths in Canada, but the persistent predominance of fentanyl in the unregulated drug supply continues to fuel overdose-related deaths [4]. To complement existing interventions, many have pointed out that providing an alternative to the unregulated toxic drug supply in the form of safer supply is critical to preventing overdose-related deaths and addressing the needs of people for whom current treatment models do not work or are not a good fit [5–11]. This approach builds on the premise that harms caused by the unregulated drug supply can be averted by providing access to a regulated drug supply [12].

Over the past few years, there has been a rapid scale up of safer supply programs in Canada [13]. Ontario is home to a dozen safer supply programs where primary care physicians and/or nurse practitioners work with other health care and service providers to embed safer supply prescribing within a broader model of care and supports for clients with complex health and social needs [14]. At the time of the study, safer supply medications in this province consisted of take-home hydromorphone tablets and directly observed slow-release morphine tablets, dosed and titrated to meet clients’ needs. Some programs required both medications to be directly observed for high-risk clients, such as those who report high-volume alcohol consumption or benzodiazepine use. Early evidence suggests that clients enrolled in safer supply programs have significantly reduced emergency room visits and hospitalizations, improved health care engagement, fewer overdoses and overdose-related deaths, reduced drug-related harms, and improved health and social outcomes [14–22].

To our knowledge, the impact of safer supply programs on injection practices has not yet been studied in the Canadian context. This gap is important to address because fentanyl is known to increase injection frequency and related health risks [23]. In fact, several international studies have documented a relationship between injection drug use (IDU), fentanyl, HIV, and HCV [24–31]. In Canada, studies conducted in Vancouver and Toronto have reported an increase in injection frequency and equipment sharing following the introduction of fentanyl in the unregulated drug supply [32, 33]. This finding is consistent with recent data from the Canadian Coinfection Cohort [34], which points to an increase in injection frequency among people who inject drugs. Finally, it is supported by a recent Canadian study that found a direct association between daily fentanyl injection, equipment sharing (i.e., syringes, cookers, and filters), and injection-related health risks [35].

Drawing on a broader set of data collected from safer supply program providers and clients in Ontario, this analysis explores changes in injection practices reported by both providers and clients. This paper summarizes the key findings and identifies potential avenues for future research on safer supply programs.

## Methods

We used data collected as part of a qualitative research study entitled *Emergency Safer supply programs: Bridging the HIV prevention, treatment, and care cascade for people who inject drugs*. For this study, service providers and clients were recruited across four safer supply programs in Ontario. To recruit providers, we sent an email invitation to the participating safer supply programs and asked for the invitation to be shared with providers. We also used snowball sampling by asking providers who enrolled in the study to share the invitation with colleagues. To recruit clients, we asked providers from the participating safer supply programs to distribute recruitment flyers to their clients. Clients who were interested in participating in the study could reach out directly to the research team during site visits or, alternatively, contact the research coordinator by phone or email. Potential participants were screened for eligibility prior to interviewing onsite or scheduling an interview time. Verbal consent was obtained prior to each interview, and a short questionnaire was completed using a secure online form. All interviews were audio-recorded, transcribed, and uploaded to MAXQDA.

The provider questionnaire included sociodemographic questions as well as basic questions focusing on professional role, years of experience, and training background. Provider interviews explored the history and implementation of each safer supply program, the process of enrolling clients and conducting initial assessments, the care provided to clients (including but not limited to safer supply prescribing), the impact of this care on clients, the successes and limitations of the program, and observations made in practice (e.g., interruption, discharge, engagement, etc.). The client questionnaire included sociodemographic questions and basic questions related to substance use, medical history, and experience of incarceration. Client interviews focused on personal history with the safer supply program, experience in the program, the impact of the program (including one question about the impact of the program on drug use, patterns of use, and overdose frequency), and differences between safer supply program and other health care services typically encountered by people who use drugs (PWUD).

The questionnaires and interview guides were not designed with the intent of documenting injection practices nor did they include specific questions to compare injection practices. Changes in injection practices were commonly mentioned when discussing outcomes of safer supply programs, suggesting a need for analyzing the data more closely and identifying changes described by clients and providers. For this secondary analysis, we extracted data where injection practices were discussed by providers and clients. The interview database was screened by K.R using a wide range of potential search terms at first and then identifying the search terms that yielded relevant content, which were inject/s/ed/ing, oral or swallow/ed/ing, smoke/s/ed/ing or snort/s/ed/ing. These terms generated a total of 315 hits across client interviews and 140 hits across provider interviews. The excerpts identified during this search were then manually screened by K.R. for relevance and further screened by M.G. and A.G. Codes were assigned to extracted data and grouped to form preliminary themes [36]. This process was completed by three members of the team (K.R., M.G., and A.G.) before themes were finalized. The final themes were reviewed and approved by the broader the study team.

## Results

Semi-structured interviews were conducted with 52 clients (see Table 1) and 21 service providers (see Table 2) across four safer supply programs between February and October 2021. We identified three core themes across client and provider interviews, each theme corresponding to a change in injection practices. The first change was a decrease in the amount of fentanyl used and a decrease in injection frequency. The second change involved switching to injecting hydromorphone tablets instead of fentanyl. Finally, the third change was stopping injecting altogether and taking safer supply medications orally. These changes in injection practices were not static or linear. They occurred and changed over time and in response to the titration of safer supply medications and overall access to care and supports.

**Table 1:** Client characteristics (*n* = 52)

	Number (*n*)	Percentage (%)
*Program site*		
Safer supply program 1	21	40.4
Safer supply program 2	15	28.8
Safer supply program 3	11	21.2
Safer supply program 4	5	9.6
Age (mean, range)	46.6	22–62
*Gender*^a^		
Man	29	55.8
Woman	23	44.2
*Indigenous*^b^		
No	42	80.8
Yes, First Nation	8	15.4
Yes, Metis	2	3.8
*Race*^b^		
White	41	78.8
Indigenous	9	17.3
Black	1	1.9
Latino	1	1.9
*Housing (past year)*		
Renting an apartment, house or condo	18	34.6
Staying with friends, family or partner	13	25.0
Staying at a shelter	7	13.5
Renting a room by the night/week/month	6	11.5
Homeless^c^	3	5.8
Owning an apartment, house or condo	2	3.8
Living in supportive or transitional group housing	2	3.8
Living in a long-term care facility	1	1.9
*Income source (past year)*		
Social assistance	51	98.1
Paid job	9	17.3
Other illegal activities	6	11.5
Other government program	5	9.6
Sex work	3	5.8
*Jail or prison*		
Ever	45	86.5
Past year	3	5.8
*HIV*		
Positive diagnosis	7	13.5
Currently taking medications	7	13.5
Undetectable viral load	7	13.5
*HCV*		
Positive diagnosis	40	76.9
*HCV medication*		
Yes, finished the treatment	20	38.5
No, never	19	36.5
Yes, currently	1	1.9
*Previous engagement with addiction treatment*		
Methadone	45	86.5
Detox	17	32.7
Live-in treatment program/facility	28	53.8
Buprenorphine	22	42.3
Outpatient	17	32.7
Treatment in jail	3	5.8
*Engagement with opioid-agonist therapy (OAT)*^d^		
No, not currently	36	69.2
Yes, currently	12	23.1
No, never	2	3.8

^a^All clients self-identified as cisgender

^b^Clients were asked to self-identify using a two-part question about ethnicity and race based on guidance provided by the Ontario Government (2016). Clients were first asked if they identified as indigenous, that is First Nations, Inuit or Metis. Then, they were asked to select which race category best described them. For example, a person who identified as Indigenous could self-report they were a different race category, e.g., white, and participants who did not identify as part of an Indigenous group in Canada could select Indigenous for their race, e.g., from an Indigenous group in another country

^c^Homelessness was defined as living on the street, abandoned building, tent/encampment, car/vehicle, outside

^d^Data are missing from two participants

**Table 2:** Service provider characteristics (*n* = 21)

	Number (*n*)	Percentage (%)
*Program site*		
Safer supply program 1	6	28.6
Safer supply program 2	9	42.9
Safer supply program 3	3	14.3
Safer supply program 4	3	14.3
*Profession*		
Nurse practitioner	5	23.8
Registered nurse	5	23.8
Physician	4	19
Other^a^	7	33.3
*Age*^b^		
Less than 35	8	38.1
35–44	8	38.1
45–59	4	20
*Gender*		
Man	13	61.9
Woman	6	28.6
Nonbinary	2	9.5
*Race*		
White	15	71.4
East Asian	2	9.5
Southeast Asian	1	4.8
Indigenous Hispanic	1	4.8
Black	1	4.8
Mixed	1	4.8
*Years worked at the site*		
Less than 6 months	1	4.8
6–11 months	5	23.8
1–2 years	3	14.3
3–5 years	5	23.8
6–10 years	6	28.6
More than 10 years	1	4.8

^a^Other included roles related to case management, administration, navigation, and coordination

^b^One practitioner did not provide their age

### Theme 1. “The more of the dilaudid i did, the less fentanyl i had to do”

The most consistent theme mentioned by both providers and clients was that safer supply medications resulted in a marked decrease in the amount of fentanyl used.

I’ve been using less. I’ve been able to control my [fentanyl] use more. I can say I’m using once a week and that’s it. Or if I want to use more, I will. Out of my $85, I will buy 2 points [of fentanyl] and spend the rest on other shit. I’ve managed to do that. (Client 1, woman, 39)

Providers also noted that by decreasing the amount of fentanyl used, safer supply medications also helped reduce the number of overdoses experienced by clients. One provider summarized what they observed in practice in this way:

I would say about seventy-five percent of people, their overdoses have reduced quite significantly, along with their fentanyl use. (…) There were people that were coming in using 25 points of fentanyl that are only using 1 point or 2 points of fentanyl every other day, which is significant. And then there are people that, regardless of their safer supply, are still actively overdosing. (Provider 1)

As highlighted by the above provider, safer supply medications resulted in a significant decrease in overdoses, but they did not completely eliminate the risk of overdosing among clients who continued to use fentanyl, even in reduced amounts, or other drugs such as stimulants. While it was evident in both provider and client interviews that the decreased use of fentanyl was a noticeable and striking outcome of safer supply programs, the decrease itself was described as gradual and nonlinear. As one client who had stopped using fentanyl explained:

I relapsed because I was hanging out with somebody that was doing it [fentanyl], and I asked them not to do it in front of me and they were still doing it in front of me, and I ended up caving in and doing some. And I’m in the process of slowing down again, down to a 1/2 point [of fentanyl] a day, pretty much, and I was at 4 points a day. So I’m in the process of quitting again. (Client 2, woman, 52)

There tended to be an inverse relationship between the use of fentanyl (i.e., frequency and amount) and safer supply prescribing (i.e., medications and dosage). In other words, clients gradually decreased their fentanyl use as safer supply medications were titrated to meet their needs. However, as both clients and providers highlighted, the process of getting to the “right dose” of safer supply medications can be complex and requires ongoing adjustments.

It was hard at the beginning but then I realized that (…) the more of the Dilaudid that I did, the less fentanyl I had to do. So it took a while to get up to the dose I’m at now (…) And I remember coming in here sick and puking, dying sick, every other day when I first started, and then going up and going up and going up [in my safer supply dosing] and finally, hey, I hit a dose and it’s “Okay, I feel comfortable with this dose. I’m at the point where I am not using fentanyl as much, I’m good”. And now it’s been a while and it’s been the same dose and I feel great. (Client 3, man, 34)

As clients decreased their fentanyl use, their injection frequency decreased and so did associated injection-related health complications—an additional positive outcome of safe supply programs identified by many providers. When asked about the impact of safer supply medications on injection-related risks (e.g., HIV, HCV, infections, etc.), one provider explained it as such:

Massively [impact of safer supply medications on injection-related risks]. We do have a lot of folks that tend to start limiting their intravenous drug use—which is great, because the more they use orally, the less they’re shooting up, the lower the risk for HIV infections, abscess, you name it, which is great. So, we do see a huge decrease in abscesses, in endocarditis, in IV use, in reinfection. It’s fantastic from that standpoint. (Provider 2)

The above quote helps illustrate another finding that came out of our analysis, which is that many clients swallowed or snorted their safer supply medications because they wanted to move away from fentanyl and eventually stop injecting. For clients who continued to inject fentanyl, safer supply medications acted as a harm reduction tool by reducing overdoses and preventing injection-related complications, including HIV, HCV, and infections (e.g., abscesses, endocarditis). They also allowed clients with poor venous access to “save their veins” and only inject when they used fentanyl as opposed to injecting multiple times a day.

I don’t inject my safer supply, I take them [the tablets] orally because my veins are shot and I save them for that one time when I do the junk [fentanyl]. (Client 4, woman, 36)

Overall, we found that as safer supply dosing increased and got closer to the “right dose,” clients gradually decreased the amount of fentanyl they injected and the frequency at which they injected. This yielded many benefits, as pointed out by the above quotes.

### Theme 2. “I’ve used the pills to inject and it got me off fentanyl for four months”

Among clients who continued to inject, some injected their prescribed hydromorphone tablets while also continuing to inject fentanyl albeit in a lower dose, or switched to injecting hydromorphone tablets only. This was often done in combination with taking slow-release morphine tablets orally. When asked if they injected safer supply medications or took them orally, one client described injecting as a stand-alone “addiction”—distinct from the substance itself.

I do both ways. I still inject. My plan is to stop injecting them [the tablets] at one point [of fentanyl], but that’s the hard thing. That’s an addiction in itself, the injecting. (Client 5, woman, 52)

Interviewer: Do you find you inject less frequently now, though, now that you’re on the safer supply than compared to before?

Yes, less. A lot less. (Client 5).

Providers noted that it was common for clients to want to inject their prescribed hydromorphone tablets because they were not ready to stop injecting and because they had remaining veins to inject into. This is important to reiterate because, as mentioned above, the decision to swallow and/or snort safer supply medications can be motivated by the need to “save existing veins” or because venous access is no longer an option. This was highlighted by many providers, including this one who said:

In the beginning most were injecting the medication and most intend to inject the medication. What’s been really interesting is—I think actually for a handful of clients, they found they didn’t actually like the sensation. There were some pins and needles that happened when they injected, and so they shifted to oral and snorting on their own. And then there’s other clients who, because their veins are just destroyed, themselves, I think, started switching from saving their injections to the one point fentanyl they might do in a day, and using their Dilaudid orally. (Provider 3)

Interestingly, as the above provider points out, injecting hydromorphone tablets was not a positive experience for all clients. Not liking the sensation of injecting hydromorphone tablets was, therefore, another reason why clients switched to swallowing and/or snorting. Similar comments were made by clients who changed their route of administration over time because hydromorphone tablets “didn’t inject well”. This client explains:

Oh, yeah. The Dilaudids—when I first started this, I’d inject them a little bit, right, and I just find—because these are injectable, and then you have the—some of—we have generic Dilaudids and stuff, too, right. What happens is they don’t inject well. They give me heartburn, right. With the Kadians, the Kadians are extended-release, right, so to bang a Kadian you’d have to crush it to powder, which is hitting it with a hammer and everything, right? But the Dilaudids, you can hit [inject] very easily. They’re almost made to be water soluble, right? And I’ve done them since I’ve been on the program, I’ve injected them a few times. And for me, it doesn’t make that much of a difference, so—and I don’t have very good veins (…) For me it just works better to take them orally. (Client 6, man, 59)

The above quote helps to illustrate that there are multiple factors influencing injection practices among clients, namely the need or desire to inject, the ability to inject (i.e., venous access), the type of safer supply medications prescribed, the availability of preferred safer supply medications (i.e., injectable medications), and clients’ personal goals. For example, some clients mentioned wanting to stop injecting and using the safer supply medication to transition away from “the needle”.

Yeah. And then, once I got my fentanyl use under control, or I was controlling it, I was able to reduce the amount all, that I was taking a day, by about half, and it’s from that now. I’m trying to switch from doing an injection into oral, because that’s my method right now, and then once I do that, I’m going to try to wean right off of them. So. My goal is to be off of opiates completely, and I think with this program, I’m going to be able to do that (...) So that’s where I’m at now. I’m trying to switch the—get off the needle. Because I think once I’m taking them orally it’ll be easier for me to wean down. I won’t be around that kind of scene any more. Needle-use scene. It’ll be easier for me to wean down. (Client 7, man, 46)

Finally, it is worth noting that continuing to inject but switching from fentanyl to hydromorphone tablets contributed to reducing overdose risks and injection-related health risks while also increasing access to harm reduction supplies and services. This was noted by providers:

For clients that are still wanting to inject, there’s a special observed room at [name of site], so they go there and be monitored while they use. (Provider 4)

As such, injecting hydromorphone tablets acted as a harm reduction intervention and as mentioned above, a pathway to “weaning off the needle” for clients who wanted to work toward that goal.

### Theme 3. “I’m starting to take the pills the right way instead of injecting them”

To reiterate, clients did not follow a linear trajectory and many shared experiences of returning to fentanyl. For some clients, this meant going back to injecting fentanyl or increasing the amount they injected while taking their safer supply medications. However, it was clear that many clients credited safer supply programs for reducing the frequency at which they injected, providing an alternative to injecting fentanyl, and allowing them to stop injecting by providing safer supply medications that are dosed properly and can be taken orally.

I talked to [name of provider] and [name of provider], and they right away put me on the safer supply and it’s been a godsend ever since. I came down from 10 points [of fentanyl] to the 1 point, and then to off it, and I take my medication religiously. I get a daily dispense. I think it’s the best thing that ever happened to me. (Client 8, man, 58)

Providers also shared stories of clients who followed a similar trajectory in meeting their goal of switching to oral hydromorphone and slow-release morphine and, in some cases, to more traditional opiate agonist treatment.

There was a lot of people changed their drug use, of the illicit supply. So, a big reduction in the amount that they were using (…) Some people stopping doing injections completely and just taking the Dilaudid orally. Or a combination, so doing an injection in the morning, right away, but then for the rest of the day taking it orally. And there were some people who transitioned off of the Dilaudid and just using the long-acting, so Kadian or methadone or suboxone. And then also things like abscesses were not as much of an issue. So just improved health outcomes. (Provider 5)

It was evident across the client and provider interviews that safer supply programs are designed to meet patient-centred goals and provide an alternative to injecting fentanyl. These programs were not driven by the goal of changing injection practices or getting clients to a place where they stopped injecting. Changes in injection practices were initiated by clients themselves because their needs were met and their personal goals were supported by the care team.

People come into the program with different goals. So, we’ve had—and it really depends on that person’s goals, right? I couldn’t say across the board. But we’ve had some people come into the program and their goal has been to titrate themselves completely off the street opioids and replace that with the prescription, and we’ve had some people who’ve had absolute success with that, and who’ve been quite content with the pharmaceutical options. And so, in their cases, I would say it has made a huge impact on their overdose risk (…) In other cases, it may not decrease the overdose risk at first, or for a while, or even ever, if that person is not decreasing their street opioid use substantially. Again, we just try to work with people’s goals. (Provider 6)

This was also mentioned by clients. It was clear that the primary goal of safer supply programs was to provide a safer alternative that worked. This process required titration of safer supply medications and meaningful involvement of clients. Any change in injection practices was an outcome of this process, as explained here:

Oh, they brought me up and up and it started levelling out and they started giving me the leeway to monitor my own pain. And it seems to be work. So I’ve told them the truth. I don’t even jam most of them [the tablets] anymore. I swallow them or I’ll snort a couple here and there, just to get that taste right away. And it seems to work. The Kadians I take mostly at nighttime, because they’re long-lasting, so when I wake in the morning I can usually stand up and walk. (Client 9, man, 60)

Clients who had stopped injecting reiterated the idea that injecting and using substances can feel like two types of “addictions.” Despite having access to safer supply medications, not all clients wanted to stop injecting, nor were they required to. This understanding was consistently reflected across client and provider interviews.

Oh, I swallow now. I swallow. If I wanted to poke, I’ll poke, right? But I don’t have to. I’m not addicted to the needle as much as I was. Because poking—that’s an addiction itself. People don’t realize that. And when they learn that, when they can separate poking from getting high, they’re able to stop poking and still get high. (Client 10, woman, 45)

As mentioned above, some clients used safer supply medications to transition out of injecting. For some, as the above quote illustrates, being able to get “high” from safer supply medications removed the need to inject. This further reinforced that in meeting clients’ needs and goals, including adequate prescribing and titration, safer supply programs can generate secondary health benefits that extend beyond reducing overdose risks. As one client explained:

Yeah. I don’t even do drugs any more. Again, I have, once in a blue moon, I’ll go out and party with some friends. I feel like I do drugs the old way again. As long as I stick to my pills, I don’t have to worry about being dope-sick. So drugs aren’t the number one priority in my mind. (Client 11, man, 37)

By preventing clients from being “dope-sick,” the “right dose” of safer supply medications opened possibilities for change. Our results suggest that injection practices are part of this change.

## Discussion

The main objective of this analysis was to explore the impact of safer supply programs on injection practices by analyzing interviews conducted with clients and providers in Ontario. Safer supply programs are not designed or implemented with the explicit goal of changing injection practices. However, the experiences of clients and providers help us understand how a structural intervention, such as safer supply, can impact other aspects of IDU (e.g., frequency of injection) and its associated health risks (e.g., HIV, HCV, etc.). As Perlman and Jordan [37] point out, structural interventions are important because “structural factors contribute potently to creating the context that renders individuals and areas vulnerable to the syndemic of [overdose, HCV, and HIV]” (p.109). These interventions work upstream, to change the “risk environment” [38, 39], rather than solely focusing on mitigating the downstream consequences at the level of the individual. Our study findings suggest that changing the “risk environment,” by providing an alternative to the toxic drug supply, creates more opportunities for risk reduction. Changes in injection practices identified in this analysis offer a compelling example.

Our findings suggest that clients enrolled in safer supply programs changed their injection practices in three intersecting ways: (1) they changed how often they injected, (2) they changed what they injected, and (3) they changed their mode of consumption (from injecting to swallowing or snorting). These findings add to existing research [16–18] by providing a more dynamic understanding of injection practices in the context of safer supply programs and further supporting the idea that safer supply can contribute to reducing injection-related health risks in addition to overdose risks [40]. We posit that safer supply programs have the potential to address disease prevention and health promotion gaps that other stand-alone downstream harm reduction interventions (e.g., needle and syringe programs) cannot address, by working upstream and providing a safer alternative to fentanyl. As Rhodes [38] reminds us, harm reduction interventions such as needle and syringe exchange programs are crucial, but their effectiveness at preventing injection-related health risks can be undermined by a particular “risk environment.” For example, if a particular shift in the drug supply results in people injecting more frequently, such is the case with fentanyl, an HIV outbreak^1^ could occur even in jurisdictions where needle and syringe exchange programs are available [38].

It is important to note, however, that not all changes in injection practices could be attributed directly to safer supply programs. We identified several indirect factors, such as poor venous access and having to inject hydromorphone tablets not intended for intravenous administration (for more on this, see study by Ivsins and colleages [17] and guidance by the British Columbia Centre on Substance Use [42]), which shaped the decision to stop injecting. Having the option of taking safer supply medications orally made this decision possible, but it is unclear if all clients who stopped injecting would have done so if they had access to a range of injectable safer supply medications and/or had better venous access. Moreover, it is unclear to what extent clients continued to inject because the safer supply medications dosage/potency was not meeting their needs, as suggested by clients who spoke of the need to supplement with fentanyl, and/or because they wanted to continue injecting. Future research should aim at exploring these nuances because safer supply programs are not intended as interventions to stop clients from injecting. If clients want to inject, they should be able to do so and access injectable safer supply medications (including injectable hydromorphone) as well as sterile supplies and supervised safer consumption services—a priority echoed in a recent report on substance use patterns and safer supply preferences of PWUD in British Colombia [43].

## Conclusion

Our findings suggest that safer supply programs could help address long-standing gaps in prevention, especially in HIV and HCV prevention [34, 37, 44, 45]. Our analysis was narrowly focused on injection practices, but we were able to identify upstream pathways of risk reduction that should be further explored. However, in order for safer supply programs to reach their full-prevention potential, we see a pressing need to expand access to a broader range of safer supply medications, including injectable *and* smokable medications, as well as safer alternatives for both opioids *and* stimulants. In the province of British Columbia, for example, the list of safer supply medications that can be prescribed as part of safer supply programs has recently been broadened to include fentanyl patches, fentanyl buccal tablets, injectable fentanyl and hydromorphone, and tablet hydromorphone. Injectable diacetylmorphine is also available in the province [46], albeit in a more limited manner. The introduction of fentanyl in the drug supply has created a new “risk environment” and changed opioid preferences [47]—matching this supply is how safer supply programs can continue to save lives and improve health.

## Data Availability

Not applicable.

## Abbreviations

IDU: Injection drug use
PWUD: People who use drugs

## Footnotes

1 A classic example of how short-acting opioids can fuel an HIV outbreak is the well-documented 2014 HIV outbreak in Scott County, Indiana (United States), a small county of approximately 4200 people facing high poverty and unemployment rates [41]. The main driver of this outbreak of 170 new HIV infections was IDU, and more specifically, the injection of oxymorphone (Opana®) [41]. This short-acting opioid was associated with a high injection frequency, as many as 20 times a day, resulting in high injection equipment sharing rates. While this outbreak was exacerbated by the lack of harm reduction services, including needle distribution programs, it remains an important example because it reflects how frequently one has to inject when using rapid-acting opioids and how unlikely it is that needle distribution programs alone can mitigate the risks associated with this practice.
